# IGF1 as a Potential Treatment for Rett Syndrome: Safety Assessment in Six Rett Patients

**DOI:** 10.1155/2012/679801

**Published:** 2012-06-13

**Authors:** Giorgio Pini, Maria Flora Scusa, Laura Congiu, Alberto Benincasa, Paolina Morescalchi, Ilaria Bottiglioni, Pietro Di Marco, Paolo Borelli, Ubaldo Bonuccelli, Andrea Della-Chiesa, Adriele Prina-Mello, Daniela Tropea

**Affiliations:** ^1^Tuscany Rett Center, Versilia Hospital, 55043 Lido di Camaiore, Italy; ^2^Neurology Division, Versilia Hospital, 55043 Lido di Camaiore, Italy; ^3^Department of Neurology, University of Pisa, 56126 Pisa, Italy; ^4^Trinity College Institute for Neuroscience, College Green, Dublin 2, Ireland; ^5^School of Medicine and CRANN, Trinity College Dublin, College Green, Dublin 2, Ireland; ^6^Department of Psychiatry, Neuropsychiatric Genetics Research Group, Trinity Center for Health Sciences, St James Hospital, Dublin 8, Ireland; ^7^Department of Brain and Cognitive Science, Massachusetts Institute of Technology, Cambridge, MA 02139, USA

## Abstract

Rett syndrome (RTT) is a devastating neurodevelopmental disorder that affects one in ten thousand girls and has no cure. The majority of RTT patients display mutations in the gene that codes for the methyl-CpG-binding protein 2 (MeCP2). Clinical observations and neurobiological analysis of mouse models suggest that defects in the expression of MeCP2 protein compromise the development of the central nervous system, especially synaptic and circuit maturation. Thus, agents that promote brain development and synaptic function, such as insulin-like growth factor 1 (IGF1), are good candidates for ameliorating the symptoms of RTT. IGF1 and its active peptide, (1–3) IGF1, cross the blood brain barrier, and (1–3) IGF1 ameliorates the symptoms of RTT in a mouse model of the disease; therefore they are ideal treatments for neurodevelopmental disorders, including RTT. We performed a pilot study to establish whether there are major risks associated with IGF1 administration in RTT patients. Six young girls with classic RTT received IGF1 subcutaneous injections twice a day for six months, and they were regularly monitored by their primary care physicians and by the unit for RTT in Versilia Hospital (Italy). This study shows that there are no risks associated with IGF1 administration.

## 1. Introduction

Rett Syndrome (RTT) is classified with autism among pervasive developmental disorders (DSM IV) and affects mostly girls (1 : 10000). The outcome of the disorder occurs through different clinical stages [1]: the onset corresponds to an interruption of growth and a decrease in the head circumference growth (stage 1) followed by a second stage with autistic features and regression of acquired skills (language, motor abilities, and purposeful use of hands), appearance of seizures, hand stereotypes, alteration in the cardiorespiratory function, and problems with the autonomic system. The third stage is characterized by a reduction of autistic symptoms, appearance of scoliosis and seizures, followed by a late regression (stage 4). There are several pathologies associated with RTT, one of which is breathing difficulties. Respiration is particularly impaired in Rett patients, and poor autonomic control is considered to play a significant role in the sudden death [2]. Respiration in RTT patients consists of periods of breath holding, hyperventilation, central apneas and Valsalva manoeuvres. Julu et al. [3] suggest that the multiple respiratory dysrythymias could be due to brainstem immaturity. In addition, the majority of RTT patients develop seizures [4, 5]. Other symptoms are muscular tone impairment, osteoporosis [6], scoliosis, self-injury behavior and sleep difficulties.

In the majority of cases RTT is caused by a mutation in the X-linked gene coding for the methyl-CpG-binding protein 2 (MeCP2), but other variants have been identified [7–9]. Since the identification of the gene [10], several groups generated mouse lines knocked out or mutant for MeCP2 [11, 12]. These mice recapitulate most of the signs observed in patients, and this property makes these mutants ideal models to study the neurobiology of the disorder and to test candidate treatments. Clinical observations and animal studies suggest that the pathology of RTT is due to an impairment in the proper maturation of brain circuitry [13, 14], placing RTT among neurodevelopmental disorders rather than neurodegenerative syndromes. A major discovery came from the research of two independent groups [15, 16] who showed that, in mice, the reactivation of the naive MeCP2 gene in the adult mutant mice was able to restore the control conditions. These reports are critical for the research of RTT because they provide evidence that, even if the normal development of the brain has been impaired by the expression of a non-functional form of MeCP2, the system still retains the ability to perform normally when the control conditions are reestablished. Another important contribution in animal research came from the study of Chang et al. [17]. The author showed that the overexpression of brain-derived neurotrophic factor (BDNF), a neurotrophin involved in brain development and plasticity, was able to counteract the deficiency in MeCP2. This study suggested that growth factors, which promote neuronal and synaptic development such as BDNF, represent potential treatments for Rett syndrome. BDNF does not cross the blood brain barrier (BBB), and therefore it has no immediate therapeutic application for RTT patients; however an alternative growth factor, insulin-like growth factor 1 (IGF1), crosses the BBB and shares similar properties and functions with BDNF. This factor is involved in the physiological development of several tissues in the body, including the brain, and, like BDNF, it modulates the maturation and plasticity of brain circuitry [18–20], it activates pathways that control synaptic function, and its pathway is modulated by MeCP2 [21]. Animal research reveals that systemic administration of the active peptide of IGF1 improves physiological behaviour and survival in MeCP2 mutant mice [14], suggesting that IGF1 can be a treatment in RTT patients. There are several arguments that support the use of IGF1 in the treatment of RTT: first, IGF1 is produced endogenously and promotes brain maturation; second, it is authorized for the treatment of IGF1 deficiencies even in childhood; third, when administered systemically, it crosses the blood brain barrier [22].

Although IGF1 has been authorized for pediatric use [23–26], it is necessary to establish whether it is safe and well tolerated in RTT patients. The most commonly reported side effects of IGF1 include hypoglycaemia, tonsillar hypertrophy, hyperplasia, and seizures. We addressed these issues in an open-label trial in six RTT patients. The drug was administered subcutaneously twice a day for 6 months. The girls were monitored each three months starting the day of the first administration until six months from the last application. We found that the treatment did not cause any side effect and it was well tolerated by the patients. This pilot study supports the use of IGF1 in patients with RTT. Further studies are planned to assess the benefits of the treatment.

## 2. Subjects and Methods

The work has been authorized by ALS 12 Viareggio and the Ethical Committee for Experimentation.

### 2.1. Patients Screening and Selection

Six girls (Subjects 1–6: S1, S2, S3, S4, S5, and S6) between four and eleven years of age with a clinical diagnosis of classic RTT were included. All subjects had the confirmed mutation in the MeCP2 gene on genetic analysis (see Table 1). Exclusion criteria were the following: patients below two years or above 13 years of age, patients not in stage three of the disorder, variants of RTT (Hanefeld variant or preserved speech variant), presence of neoplasia, and medical history of hypoglycemia. The girls older than 13 were excluded to avoid the risk of acromegaly associated with IGF1 treatment [27]. Although the IGF1 can be safely administered to girls older than two years, only families with girls older than four years volunteered to participate to the trial. Families gave written consent for their participation in the study before starting the treatment. Initially, 40 patients were screened and six girls were selected according to the criteria described above.

### 2.2. IGF1 Administration

IGF1 (Mecasermin-Increlex*) was administered subcutaneously twice/day for six months. The caretakers were trained to perform the injections at home. For the first and the last weeks the drug dosage was 0.05 mg/Kg; during the remaining periods the dosage was 0.1 mg/Kg. The dosage is the same used in the IGF1-deficiency treatment. The maintaining dosage is the one approved for treating IGF deficiencies in children [24, 26]. Possible side effects are hypoglycaemia (headache, dizziness, nausea, and perspiration), tonsil hypertrophia, and local irritation and pain at the injection site. We planned five clinical sessions in the hospital: Day 0 (D0), where families and caretakers were informed about the treatment and trained to perform the injections and to measure glucose levels. For the initial administration, each patient was hospitalized for 1 day and the families signed the consent form for treatment. None of the patients reported any of the possible side effects of IGF1 at the time of hospitalization. The caretakers were trained to perform the daily injections and were provided with the kit to measure glycemia levels. During the first hospitalization, the following parameters were measured: auxiologic and ematic values, cardiac activity (ECG), breathing, and EEG and bone density. In addition, the patients were clinically evaluated. The same parameters were measured during each of the following sessions, with the exception of bone density, which was evaluated after one year. The caretakers were asked to fill a journal reporting sleep activity, eating, and any observed abnormal behaviour. 

### 2.3. Patients' Evaluation

The patients were evaluated clinically and neurologically, and the cardiorespiratory parameters and brain activity were investigated with the Neuroscope (Medifit Instruments Ltd., London, UK) [3, 28]. Autonomic and cardiorespiratory function indexes were recorded noninvasively and synchronously with EEG and video over a one-hour period. The autonomic parameters included cardiac vagal tone, heart rate, transcutaneous blood gases, and respiratory patterns. Following analysis of the recording each patient was assigned to a specific cardiorespiratory phenotype according to her respiratory dysrhythmia. Thirteen abnormal, awake breathing rhythms have been identified in the RTT population and categorised into Feeble, Forceful, and Apneustic type of breathings. The RTT population can be subdivided into three groups of Breathers corresponding to these breathing types. They constitute three unique cardiorespiratory phenotypes with different levels of blood gases, autonomic tone, physical features, clinical complications, and idiosyncratic responses to drugs. Rational approaches to clinical management are different and unique for each phenotype [29]. 

For clinical evaluation, we used the following scales: International Severity Scale (ISS) Clinical score [30], Clinical Global Impression (CGI), Severity [31], and a domestic scale developed in our hospital for the evaluation of RTT patients (PBZ scale, data not published). ISS consists of 21 items evaluating the typical RTT characteristics, divided in five subscales: Growth and Development, Muscolo-Skeletal appearance, Movement, Mental-Cortical, and Brainstem-Autonomic. Each item score ranges from 2 to 0 as follows: 2: severe abnormalities, 1: mild abnormality, and 0: no abnormality. CGI-S measures the severity of symptoms in mental disorders based on a seven-point scale, “1” meaning very much improved to “7” meaning heavily affected. 

Blood and urine samples were collected, and we evaluated bone density, EKG, EEG, and respiration abnormalities. For the first drug administration the patients were hospitalized for 24 hours. The following administrations were performed at home. Follow-up appointments occurred at 90 days (D90), 180 days (D180) from the first administration, and then at 90 and 180 days following the last administration (D270 and D365, resp.). Clinical evaluation and blood tests were repeated as for D0, and ECG, EEG, repeated. The drug was gradually discontinued, and during the last week of treatment the dosage was reduced to 0.05 mg/Kg/die (as for the first week).

### 2.4. Measurement of Brain Activity

EEG measurements were conducted with electrodes in the “10–20 scheme” approved by the International Society of Electroencephalography and Neurophysiology. The recordings lasted 1-2 hours. The analysis was performed in 28 minutes as it is the minimum time of stable recording.

EEGs were sampled at 128 kHz (samples/sec) for a minimum duration of 28 minutes each. During the recording sessions the girls were awake. In this analysis, we considered two groups of RTT patients: one group did not receive neither IGF treatment nor a placebo, while the second group received the IGF1 treatment. The groups were homogeneous for age and clinical features. The amplitude and frequency of theta and delta waves were evaluated as they are the most relevant for RTT [32].

Analysis was conducted using a custom-made software in MATLAB that allows the investigation of frequency and time domains. The script consists of a fast Fourier transform (FFT) function that analyzes the signal divided in five sec bins extracting the frequency, the time, and the amplitude domains. Peak and mean frequencies in the delta range (1–4 Hz) and theta range (4–12 Hz) and their associated amplitude were separately averaged over the whole signal and grouped in bins to be used as comparative variables between and within the groups. Delta and theta ranges frequencies and amplitudes were chosen as main parameters because of the relevant correlation of these brain oscillations with cognitive performance. In particular, the theta oscillations strongly correlate with memory and learning performance. 

For statistical comparison of groups across different sessions, we used the nonparametric Wilcoxon test, which considers the datasets containing observation for the same sets of subjects. In addition, we report data for single subjects.

Parents were asked for observed side effects at every visit. 

## 3. Results

### 3.1. Patients' Selection and Initial Assessment

Six RTT patients between four and eleven years old with the classical form of RTT were treated for this study (see Table 1). The subjects studied are the following: S1: 4.8 years, MeCP2 mutation: deletion of exons 3 and 4, with a severity of illness of 4 (CGI); S2: 8.10 years, MeCP2 mutation: T158M (missense), severity of illness of 6 (CGI); S3: 5.8 years, MeCP2 mutation: R133C (missense) severity of illness of 3 (CGI); S4: 5.9 years, MeCP2 mutation: C13insGCCGC in exon 1 (frameshift), severity of illness of 3 (CGI); S5: 4.7, MeCP2 mutation: R270X (nonsense), severity of illness of 5 (CGI); S6: 10.8, MeCP2 mutation 1055 del 12 + 1157 del 44 (frameshift), severity of illness of 3 (CGI). For a summary of subjects and mutations see Table 1. At the start of IGF1 administration, S1, S2, and S3 were already under treatment for epilepsy with valproic acid at the dosage of 20 mg/kg/die.

### 3.2. Blood Parameters

**Figure 1:**
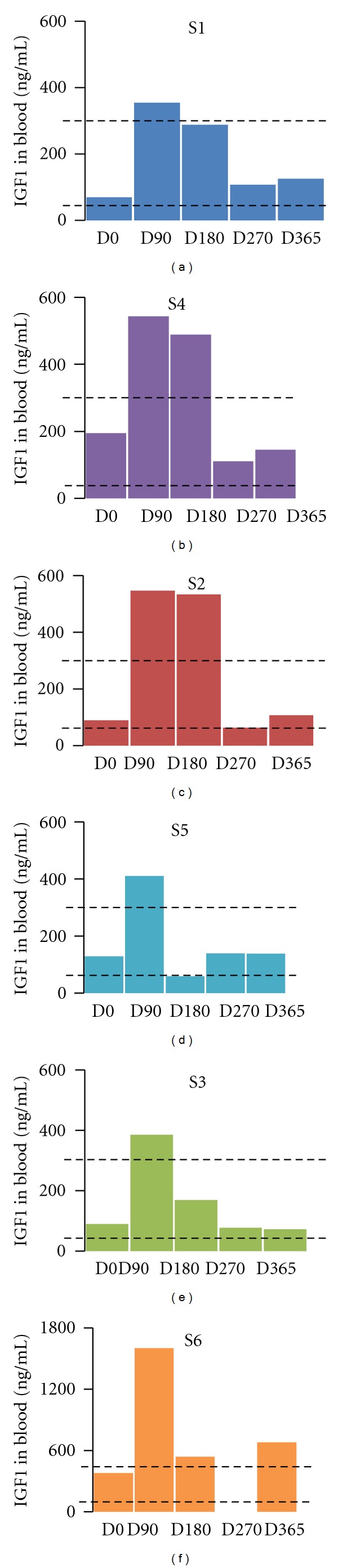
IGF1 serum levels during the treatment. IGF1 levels in the blood were tested at each hospitalization: D0 (first day of treatment, before IGF administration), D90 (90 days after the first IGF1 administration), D180 (180 days after the first IGF administration, last day of IGF1 treatment), D270 (270 days after the first IGF administration), and D365 (365 days after the first IGF administration). The dotted lines represent the normal range of IGF1 in each patient. The amount of IGF1 is expressed in ng/mL and reported for each patient, and it is color-coded: S1: blue, S2: red, S3: green, S4: purple, S5: cyan, and S6: orange. The data for S6 at D270 is not available. The serum levels of IGF1 rose for each patient during the treatment and returned back to control levels after the end of the treatment.

During hospitalizations the blood parameters were measured. In particular, blood count (leucocytes, haematocrit, haemoglobin, neutrophils, and lymphocytes) revealed no significant changes during the treatment while serum levels of IGF1 increased—as expected—during the treatment and went back to baseline values after the end of IGF1 administration (Wilcoxon test, *P* value = 0.03). The initial endogenous levels of IGF1 were within the age range for each patient (Figure 1). Glucose and growth hormone levels stayed within the reference values in each patient, while insulin level dropped below the expected level in S1 and S2 during IGF1 administration.

Blood gas values at baseline were abnormal prior to commencing treatment, consistent with the respiratory pathology of RTT, where patients present irregular episodes of hyperventilation, breath holding, and apneas. Changes in venous blood gas parameters (pO_2_, pCO_2_, pH, O_2_Hb, HHb, CHCO_3_, and SatO_2_) revealed changes in all patients during IGF1 treatment, but no definite pattern is visible and statistical analysis reveals no significant differences between sessions. The specific values are reported in Table 2: pO_2_ was initially normal for S1, but it reached higher values after IGF administration and returned within the range after the suspension of the drug. Conversely, S3, S4, and S5 had always regular values for pO_2_, and so it was for S2, with the exception of D270. For S6 the recorded values were always above the range. pCO_2_ measurements were always normal for S1, with the exception of D90, when they went below the limits. S2 had an abnormal pCO_2_ values before starting the treatment, they went in the normal range during the treatment and returned below the limits after the suspension of the drug. S3 had normal pCO_2_ values below the limits at the beginning of the treatment but the values were in the normal range in subsequent sessions. S4 had normal pCO_2_ values at D0 and D90 and the values went above the range after D180. For S5 the pCO_2_ value was always above the limits while it was constantly in the normal range for S6. The pH values were in the normal range in the majority of cases with the exception of S2 at 270, S4 at D180 and D270, and S5 at D90. The measurements of O_2_ Hb and Hbb were normal for all the patients across all the sessions and therefore they are not shown in Table 2. Conversely, the CHCO_3_ values were altered in the majority of cases with the exception of S6, who had always normal values. The SatO_2_ initially was abnormally high for S1 and returned to normal values after D270, while for S2 the measurements were always normal with the exception of D90, when it became abnormally low. S3 and S4 had initial values above the range, but they went back to normal in the following sessions. For S5 and S6 the SatO_2_ values were constantly abnormal (below the range for S5 and above the range for S6).

### 3.3. Growth Parameters

**Figure 2:**
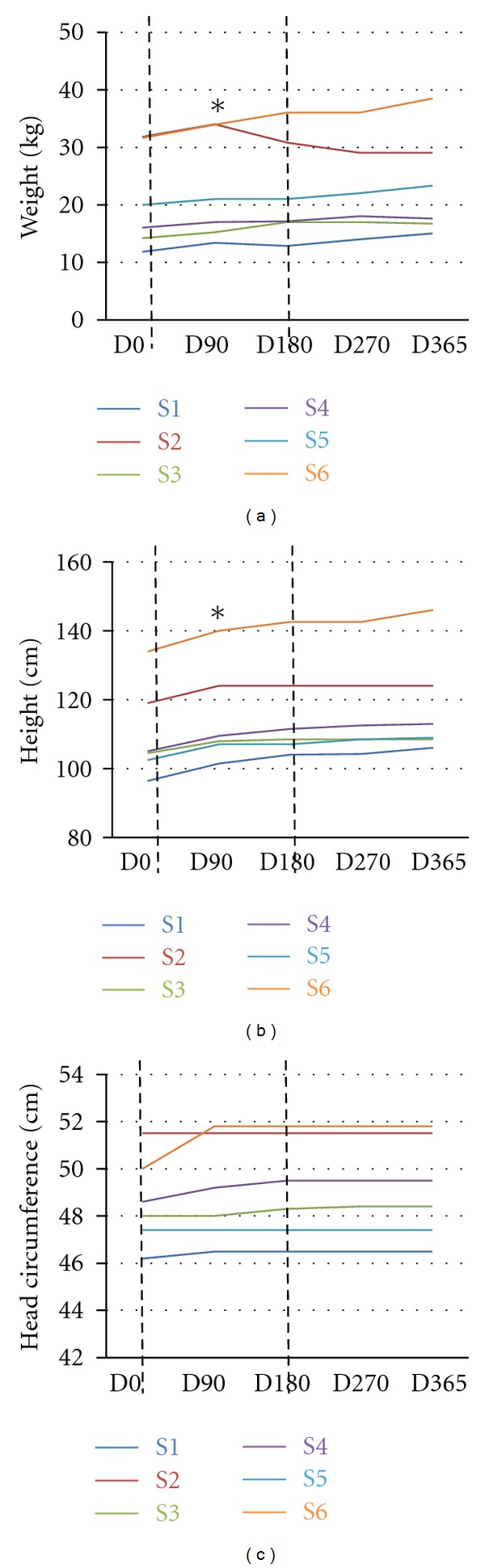
Growth parameters progression during IGF1 treatment. The figure reports the increase/decrease in weights (a), height (b), and head circumference (c) in each patient for one year, starting from Day0, D0 (first day of treatment, before IGF administration). The measurements were taken at D90 (90 days after the first IGF1 administration), D180 (180 days after the first IGF administration, last day of IGF1 treatment), D270 (270 days after the first IGF administration), D365 (365 days after the first IGF administration). The dotted lines represent the start (D0) and the end (D180) of the treatment. Each patient is color-coded: S1: blue, S2: red, S3: green, S4: purple, S5: cyan, and S6: orange. (a) Changes in body weight are expressed in kgs. There is an increase in body weight for each patient, with the exception of S2, who after D90 shows a decrease in body weight. The increase between D0 and D90 is significant (Wilcoxon test). (b) Changes in height are expressed in cms. The patients show a significant increase in height between D0 and D90 (Wilcoxon test). (c) Changes in head circumference are expressed in cms. The patients show an initial—but not significant—increase in head circumference between D0 and D90.

We measured the growth parameters: body weight, height, and head circumference (Figure 2). In all of the patients we observed an increase in body weight, with the exception of S2, who had a reduction in the body weight following 3 months of treatment. Across all patients, there was a significant increase in body weight between D0 and D90 (Wilcoxon test, *P* value = 0.03) with the exception of S2, who was overweight at the beginning of the treatment, and she reduced body weight. The growth trend continued even after the treatment was suspended, and this is true also for S2 who continued to lose weight, although she remained overweight despite the IGF1-driven weight loss.

Patients showed a significant increase in height between D0 and D90 (Wilcoxon test, *P* value = 0.03) that continued even after the end of the treatment. Head circumference showed a trend of increase (though not significant) in all the patients with the exception of S5, who was unchanged (Figure 2). 

It is important to stress that even if there was an increase in the parameters examined, the growth remains impaired to the development of normal age-matched girls.

### 3.4. Glycemia Measurements

Each family received a kit for the detection of blood level of glucose, and the glucose concentration in the blood was measured by the caretakers every day for the after 60 minutes from the IGF1 administration, in addition, hypoglycaemia was evaluated as part of blood tests during periodic hospitalization. After few weeks, the glycaemia was measured only if one of the symptoms was observed (dizziness, sweating, headache, fatigue, nausea, irritability, difficulty concentrating, and tachycardia).

**Figure 3:**
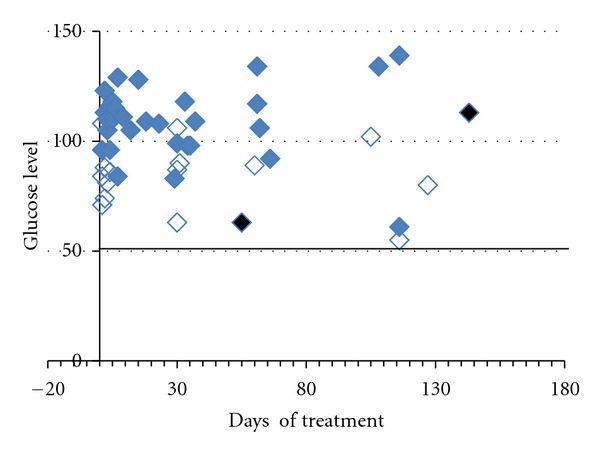
Glycemia values in Subject 1. Glucose blood levels were registered by the caregiver and recorded in the journal. The measurements were taken with a home device that the caregiver was trained to use on D0. On the *X*-axis are reported the days when the single measurements were taken, on the *Y*-axis are reported the glucose levels. For each measure the label shows the condition: blue filled diamond: full stomach, empty blue diamond fasting: and black filled diamond: vomit episode. The horizontal black line represents the threshold below which it was considered hypoglycaemia (glucose value 50).

Hypoglycaemia was cautiously defined as blood levels of glucose below 50 mg random. None of the patients went below the set threshold; however, one patient (S1) was reported to have a glucose value of 55 at 118 days from the beginning of the treatment (Figure 3). The measurement followed a period of vomiting and fasting. Treatment was suspended and restarted after 24 hours, following several normal measurements of glucose level.

### 3.5. Seizures Outcome during IGF Treatment

**Figure 4:**
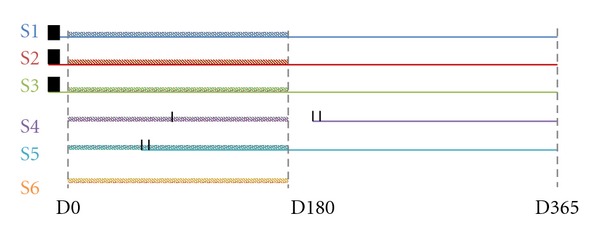
Seizures outcome during IGF1 treatment. The figure shows the outcome of seizures during one year starting with D0. The dotted lines represent the start (D0) and the end (D180) of the treatment. Each patient is color-coded: S1: blue, S2: red, S3: green, S4: purple, S5: cyan, and S6: orange. The black vertical lines represent seizures episodes. The color-coded horizontal straight lines represent the therapy for seizures (valproic acid), while the dashed color-coded lines represent the IGF1 treatment that lasted from D0 to D180. Patients S1, S2, and S3 had seizures before the start of IGF1 treatment, and they were simultaneously treated with valproic acid and IGF1. S6 did not develop seizures. S4 had one single episode of seizures around D90, and the treatment with valproic acid started only after D180, when the seizures become frequent. S5 showed frequent seizures around P90 and the treatment with valproic acid started immediately.

Some of the girls (S1, S2, and S3) in this study had seizures before starting the IGF treatment, and they were regularly treated with valproate. During crisis, S1 had staring eyes and muscular rigidity; S2's crisis started with cyanosis, followed by lateral limbs hypertonicity, pallor, loss of consciousness, and hypotonia. S3 had shaking, hypotonia followed by muscular rigidity, staring eyes, guttural sounds, and sleep. IGF1 treatment was administered alongside regular antiepileptic medication (AED) as described without changing the therapy for seizures. Seizure frequency and AED therapy remained unchanged, suggesting no interaction with valproate. S5, S4, and S6 did not report seizures before the IGF1 treatment. One of them, S6, never showed seizures, while S4 and S5 showed seizures during the IGF treatment. S4 had a single episode of seizures after three months from the start of the treatment, characterized by jaw clonic jerks and guttural vocalization for less than one minute, and AED was not indicated. Following discontinuation of IGF treatment, S4 had repeated and prolonged episodes of the described seizures and valproate was commenced.   S5 developed seizures two months after the start of IGF1 therapy and was commenced on valproate (Figure 4).

### 3.6. Cardiorespiratory Activity

Patients with classic RTT have impaired cardiorespiratory function. Four out of six patients presented with superficial breathing and apneas (S4, S2, and S6). One patient, S3, presented with “enhanced phenotype,” that is, tachypnea and hyperpnea. S1 had an alternating pathology of apneas and tachypneas with no particular prevalence of one. In addition, three of the six patients, S4, S2, and S3, had a high prevalence of Valsalva manoluvre.

Evaluation of cardiac function included measuring R-R distance on ECG, heart rate, and vagal tone. No significant changes in these measurements were detected during IGF1 treatment. 

**Figure 5:**
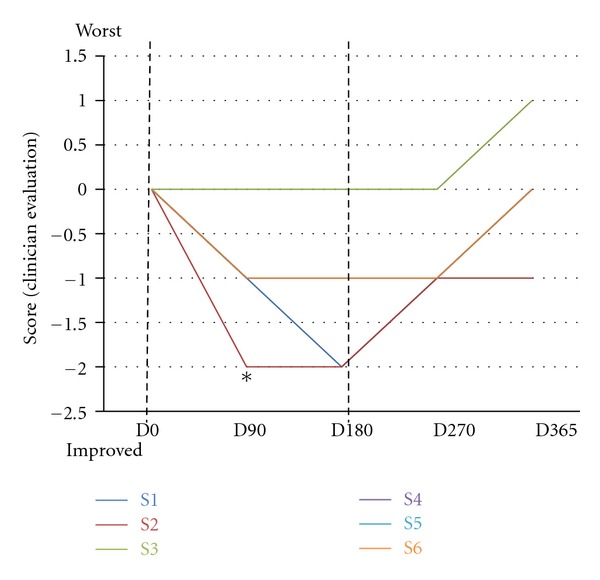
Clinical assessment of IGF1 treatment on breathing. The graph reports the clinical evaluation of respiratory activity at each visit compared with the previous observation. D0 (first day of treatment, before IGF administration), D90 (90 days after the first IGF1 administration), D180 (180 days after the first IGF administration, last day of IGF1 treatment), D270 (270 days after the first IGF administration), and D365 (365 days after the first IGF administration). The dotted lines represent the start (D0) and the end (D180) of the treatment. Each patient is color-coded: S1: blue, S2: red, S3: green, S4: purple, S5: cyan, and S6: orange. The starting score at D0 was 0, and it was decreased or increased if the respiration improved or worsened, respectively. All the patients, with the exception of S1, showed an improved respiratory activity during IGF1 treatment. The improvement across all the patients is significant between D0, and D90 (Wilcoxon test). After the IGF1 administration was interrupted, respiration worsened for all the patients. The worsening occurred in the majority of cases immediately after the interruption, for two patients (S1 and S6) after D270.

To evaluate the breathing activity, during the hospital visits, we monitored the respiration for one hour, we performed a clinical evaluation of the respiratory activity, and we measured the following parameters: breath holding, apnea, Valsalva's manoeuvre, and normal respiration. The measurements were used for the clinical evaluation of the breathing activity, which is represented in Figure 5. The effects on the single parameters and blood gas analysis (pO_2_, pCO_2_, and pH) were highly dependent on the patient and did not show any significant trend (Table 2). Clinical assessment, after evaluation of the general respiratory pattern, suggested an improvement during IGF1 treatment with a regression after the end of the treatment (Figure 5). We compared the clinical assessments scores for each patient between consecutive sessions and we found a significant improvement between D0 and D90 (Wilcoxon test, *P* value = 0.04). 

### 3.7. Bone Density

**Figure 6:**
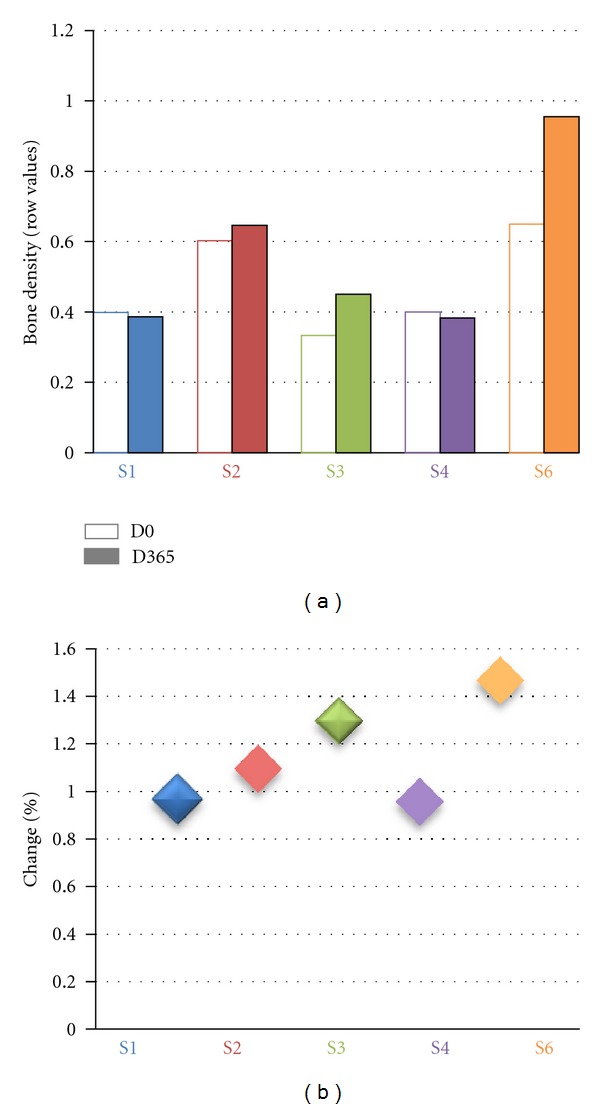
Effects of IGF1 treatment on bone density. (a): row values for bone density for each patient at D0 (empty histogram) and D365 (filled histogram). D0 (first day of treatment, before IGF administration), D90 (90 days after the first IGF1 administration), D180 (180 days after the first IGF administration, last day of IGF1 treatment), D270 (270 days after the first IGF administration), D365 (365 days after the first IGF administration). The dotted lines represent the start (D0) and the end (D180) of the treatment. Each patient is color-coded: S1: blue, S2: red, S3: green, S4: purple, and S6: orange. (b) Percentage of change between bone density at D365 versus bone density at D0. The majority of patients showed a ratio of 1, while for S3 and S6 the ratio was bigger than one showing an increase in bone mass after the treatment.

We then measured the effect of IGF1 treatment on bone structure [31]. Bone density was measured at baseline and after one year from the beginning of the treatment (D365). The readings for S5 are not available for technical reasons. S3 and S6 had increased bone density following treatment. Bone density was unchanged in the remaining subjects (Figure 6).

### 3.8. Evaluation of Brain Activity—EEG

**Figure 7:**
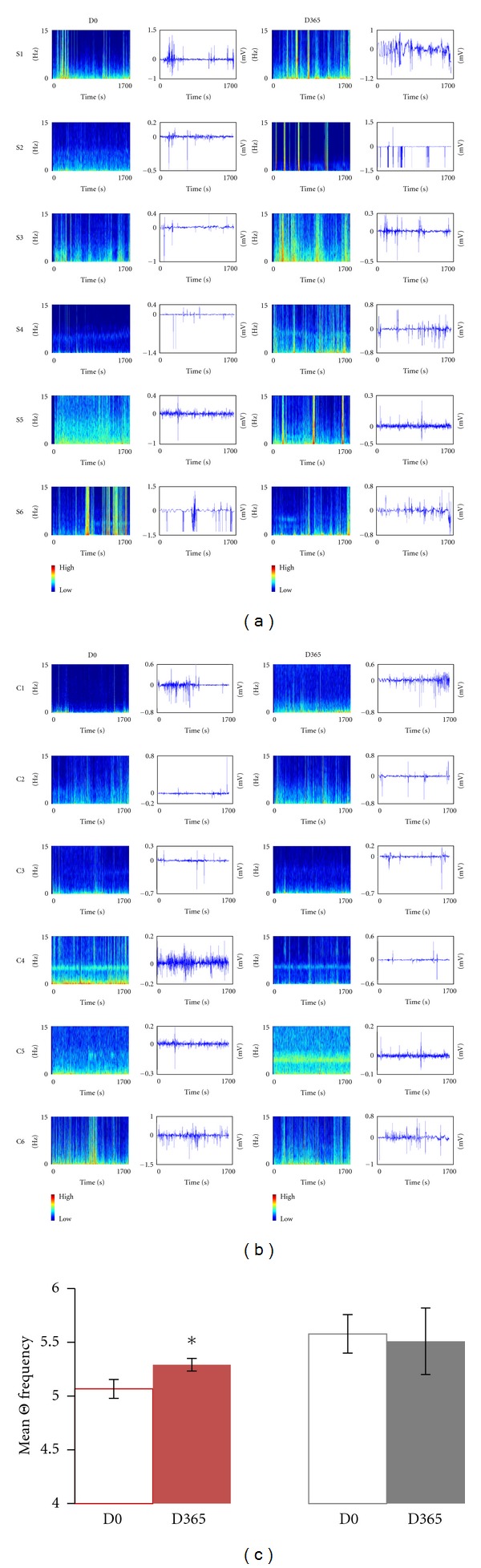
Effects of IGF1 treatment on brain activity. (a) Spectrum of frequencies (left) and EEG trace (right) for the site C4-T4 in all the treated subjects S1–S6 at D0 and D365. (b) Spectrum of frequencies (left) and EEG trace (right) for the site C4-T4 in all the untreated controls C1–C6 at D0 and D365. The color bar on the right shows intensity scale. (a) and (b): Frequencies spectrum *Y*-axis (blue plots) ranging between 0 and 15 Hz; *X*–axis ranging between 0 and 1700 sec. EEG traces *Y*-axis (white plots) ranging between −1.5 and +1.0 mV; *X*-axis ranging between 0 and 1700 sec. (c) Quantification of theta frequency (site C4-T4) in all the treated (T-red) and all the nontreated (NT-grey) RTT patients at D0 (non filled) and D365 (filled). There is a significant increase in theta frequency between the treated but not the untreated patients (Wilcoxon test).

Patients were assessed for brain activity on D0 and after one year (D365). Ten electrodes were placed on the scalp and the following signals recorded: Fp2-C, C4-T4, T4-O2, O2-C4, Fp1-C3, C3-T3, T3-O1, and O1-C3. The treated patients were compared to a group of six untreated RTT patients (age matched and with comparable RTT clinical stage). We considered the signal of each derivation, and we evaluated both the amplitude and the spectrum of frequency for each derivation (Figure 7). We focused on delta and theta signals as they are more relevant for RTT [32]. The analysis of amplitude revealed that the mean delta amplitude was not different between treated and not treated patients at the beginning and at the end of the treatment and that IGF1 did not cause any change in this parameter. The frequence of delta waves remained unvaried for both treated and untreated patients between the beginning and the end of the treatment.

The amplitude of teta waves was not significantly different between D0 and D365 (Figure 7). The frequence of theta waves was significantly higher in treated versus untreated patients at D365 for the electrode C4-T4 (Wilcoxon test, *P* value = 0.04) (Figure 7). 

### 3.9. Clinical Evaluation according to International Severity Scale

**Figure 8:**
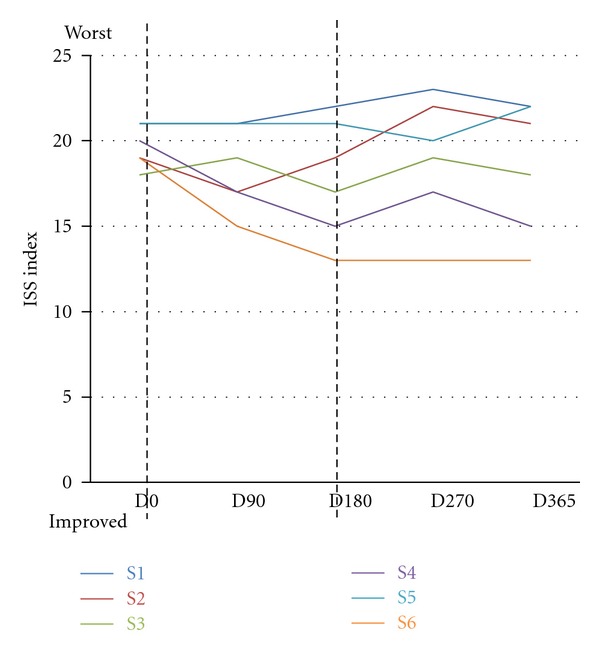
Effects of IGF1 treatment on International Severity Scale (ISS). The graph reports the clinical evaluation of the parameters of the International Severity Scale (ISS). D0 (first day of treatment, before IGF administration), D90 (90 days after the first IGF1 administration), D180 (180 days after the first IGF administration, last day of IGF1 treatment), D270 (270 days after first IGF administration), and D365 (365 days after first IGF administration). The dotted lines represent the start (D0) and the end (D180) of the treatment. Each patient is color-coded: S1: blue, S2: red, S3: green, S4: purple, S5: cyan, and S6: orange. At each visit the patients were evaluated and the score for the ISS annotated. An increase in the score shows a worsening of the conditions, while a decrease in the ISS score represents an improvement. During IGF treatment (D0–D180), half of the patients showed an improvement in the ISS score. Between D180 and D270, the ISS score increased for all the patients with the exception of S5 and S6. At D365, S6 and S4 had an improved ISS, score, while S3 had the same score as that at D0. S5, S2, and S1 had a higher ISS score at D365 than at D0. The improvement in ISS score is not significant considering the entire cohort of patients.

During hospital evaluation the patients were scored for the parameters of the International Severity Scale (ISS). This scale takes into account parameters of growth and development (growth, body weight, and height), locomotor apparatus (muscle tone and spinal column), locomotor ability (deambulation, stereotypes, and purposeful use of hands), cortical functions (cognition, epilepsy, and speech), and autonomic functions (respiration, circulation, digestive system, and sleep). The lower the score on this scale, the better the abilities. At the end of IGF treatment (D180) we observed a reduction (improvement) in the ISS score for the patients S6, S4, S3, and S2 (Figure 8), while for S5 and S1 we observed no changes or even a slight worsening. After six months from the end of IGF1 treatment (D365) there was an improvement in the ISS score for S4 and S6, while for S3 the ISS score was the same as that at D0 and for S1, S2, and S5 was higher (Figure 8). The improvement in ISS parameter was not significant considering the whole cohort of treated subjects.

### 3.10. Sleep, Nutrition, Language, and Cognition

At the first visit the families were given a journal for filling in information regarding sleep, nutrition, abnormal behaviour. According to the annotations non particular changes occurred during or after IGF1 treatment in the sleep and eating behaviour of the patients. 

No changes were observed in the language abilities although parents and caregivers report an increase in the communication abilities, that is, the girls were more able to communicate their intentions/needs. These improvements are in line with a more general improvement in cognitive abilities and attention that has been reported for all the patients even after the end of the treatment. Both teachers and family members report that the patients were more able to engage with the environment and to interact with friends/family members. However, an appropriate test is required for assessing the cognitive benefits of IGF1. Such measurements are beyond the purpose of this study.

### 3.11. Side Effects and Parents Assessments

**Figure 9:**
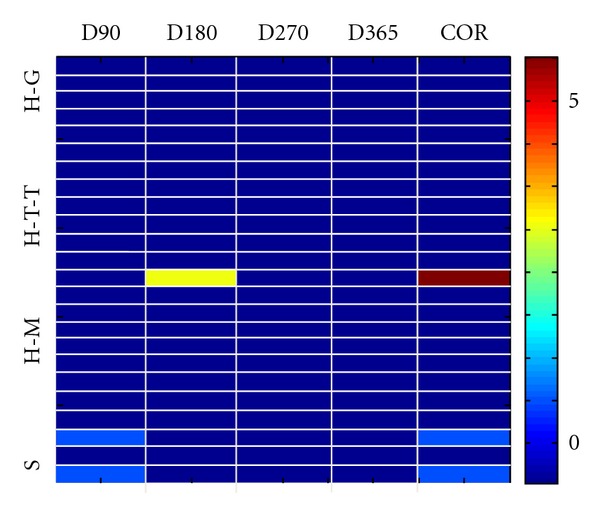
IGF1 treatment is safe and tolerated by patients. Representation of the possible side effects of IGF1 in all of the treated patients: hypoglycemia (H-G), tonsillar hypertrophy (H-T-T), mammary hyperplasia (H-M), and seizures (S) at different times after IGF1 administration (D90, D180, D270, and D365) and correlation with IGF1 administration in all the treated patients. In the graph is reported the score for all the possible side effects in each subject and the correlation between the symptom and IGF1 administration (colorbar values from 1 to 5). The graph shows that only one patient—between D90 and D180—presented moderate mammary hyperplasia (score 3). This event is highly correlated to the IGF1 treatment (correlation score 5, values on the right) but disappeared spontaneously and without the administration of additional therapy. Two additional patients showed seizures during the treatment (score 1) but these are not associated with IGF1 treatment (correlation score 1).

No major side effects were observed in any patient; however, we report mammary hyperplasia for S1, first on the right side, after 40 days from the beginning of the IGF treatment, then on the left side (Figure 9). Ultrasound analysis did not reveal additional morbidity. The hyperplasia regressed on both sides within two weeks without changes to the IGF treatment. 

We asked the parents after one year if they would repeat the IGF treatment. Four families out of 6 answered “Yes,” while S1 and S5 families would not repeat the treatment.

## 4. Discussion

RTT is a rare neurodevelopmental disorder that affects mostly girls (1 : 10000) and has no cure. Because there are several pathologies associated with RTT, often the treatments have been oriented to control specific symptoms and clinical trials have been carried out during the years to assess the effects of several drugs for treating sleep efficiency, respiratory dysfunctions, or seizures [33–35]. However, the evidence is that benefits from tested therapies are limited. The discovery of the gene associated with RTT syndrome and the generation of animal models enormously improved the knowledge of the disease. Animal studies showed that brain circuitry in MeCP2 mutant mice is immature [14] and that function can be restored even in the adult animal [15]. In addition, they showed that growth factors involved in brain maturation and plasticity are potential candidates for ameliorating the symptoms of RTT [14, 17].

So far IGF1 is the only treatment that affects multiple symptoms of RTT in animal models and has the potential to be administered to human patients considering that it is already approved for pediatric use [36] and its ability to cross the blood brain barrier [22]. 

In this study we ran a pilot trial in six subjects to assess the safety and tolerability of IGF1 treatment in RTT patients. Although we are aware that the small sample and the open-label nature of the trial are not suited to establish the benefits of IGF1 treatment in RTT syndrome, this pilot study is purely aimed to assess the safety and tolerability of IGF1 in RTT patients. This assessment is critical for future studies aimed at determining the benefits of IGF for RTT in a larger cohort of subjects. The patients were homologous for age range and stage of the disease; however, the symptom severity was slightly different between them. The main possible side effects of IGF1 treatment are hypoglycaemia, tonsillar hypertrophy, hyperplasia, and seizures (Figure 9). We found that only one patient developed mammary hyperplasia. This event was correlated to the IGF1 treatment, but regressed spontaneously and without any additional therapy or interruption of IGF1 administration. Two patients developed seizures during the treatment. Although we cannot exclude that IGF1 treatment influenced the appearance of seizures, 80% of RTT patients at that age develop seizures, and therefore the onset can be due to the normal phenotype of RTT. In support to this hypothesis we note that the other girls that received IGF1 treatment together with AED did not show an increase in seizures, nor they had to adjust the AED dosage. 

In addition, we checked the safety and tolerability of IGF1 treatment on other parameters: blood parameters, cardiorespiratory activity, bone density, brain activity, motor abilities, and eating and sleep disorders. The blood parameters did not show any relevant change between D0 and the following sessions for the majority of patients, the only exception being the amount of IGF1 in the blood which increased during the treatment and went back to control levels after D180. The values of gas parameters were highly variable for each patient and each session, and they fail to show any trend with IGF treatment: IGF does not increase or decrease a specific parameter. We conclude that IGF1 administration has no effect on gas parameters. Patients received clinical evaluation of ECG and respiratory activity for each session. There was no alteration in the cardiac activity, while the respiration changed for each session and each patient (which is consistent with the clinical features of the condition). For logistic and technical reasons we were not able to measure the breathing activity profile for all the sessions; however, clinical assessment show high variability across time and patients. Overall, the treatment did not reduce the respiratory performance and clinical evaluation showed a significant improvement of breathing during IGF1 treatment. Since IGF1 is involved in bone formation [37] and bone density is reduced in RTT patients, we expected to see an effect on this parameter. Indeed two patients (S3 and S6) showed an increase in bone density. For the other patients no relevant change was observed. For the analysis of brain activity, we measured EEG analysis at D0 and D365 in treated patients compared to untreated RTT patients of the same age range and stage of disease. There is a significant increase in the mean frequency of theta waves before and after the treatment for the location C4-T4, suggesting that the IGF1 treatment increased the frequency of theta waves in the treated patients. Since theta waves have been associated with learning processes, these results are consistent with the increase in cognitive abilities observed for all the treated patients. However, the observed improvement in cognitive abilities was subjective, additional studies are requested to confirm this hypothesis. The motor abilities remained unvaried for three of the patients S3, S2, and S5, while they improved for S4, S3, and S6. Of note the patients that showed an improvement in motor skills were already able to move with support, while S1, S2, and S5 required wheel-chair support. Families report no changes in the sleep/eating pattern during IGF1 treatment. There is a significant increase in the growth parameters; however, the percentiles did not change significantly within the range of normal development, suggesting that IGF1 treatment improves the conditions of the patients but does not restore the normal conditions. 

## 5. Conclusion

The data show that IGF1 administration is safe and well tolerated by the tested patients and can be administered to RTT patients with consistent monitoring of the risk parameters. One subject developed bilateral mammary hyperplasia but it regressed spontaneously without administration of additional therapies or interruption of IGF1 treatment. The appearance of seizures in two of the subjects during the treatment is in line with the seizure outcome in RTT patients in the considered age range and therefore it is unlikely to be determined by IGF1—although we cannot exclude it. This observation is supported by the fact that the subjects that were exposed to IGF1 being previously treated with AED did not experience an increase in seizures nor required any adjustment of the antiepileptic medications. 

All the families and caretakers observed an improvement in the cognitive abilities of the girls and in the interactions with the surrounding environment. However, we could not quantify this improvement. We plan to investigate this aspect with the appropriate equipment in further studies. Interestingly, the patients with the less severe symptoms reported improvements in motor abilities (S3, S4, and S6) and in some cases the improvements were retained even after the end of the treatment. We observed no relationship between the effects of IGF1 treatment and the specific MeCP2 mutation, although the number of patients treated was too low to establish any correlation. Results from this study suggest that the IGF1 treatment is well tolerated by RTT patients and does not present major and/or permanent side effects. Three out of six girls treated reported benefits, although a bigger double-blind placebo-controlled study is requested to quantify the efficacy of the treatment.

## Figures and Tables

**Table 1:** Selected patients for the study. This table reports the patients in the study, with their specific mutation, D0 and age at the beginning of the treatment. In addition, it reports the gravity of the disease (7 heavily impaired, 1 mildly impaired).

ID	D0 (Age)	Mutation	Severity (1–7)
S1	4.8	Del exons 3 and 4	4
S2	8.1	T158M (missense)	6
S3	5.11	R133C (missense)	3
S4	5.9	C13ins in GCCGC in exon 1 (frameshift)	3
S5	4.7	R270X (nonsense)	5
S6	10.8	1055 del 12 + 1157 del 44 (frameshift)	3

**Table 2:** Gas parameters. This table reports the measurements of gas parameters for each patient at D0 and following hospitalization. NR: not registered. NA: not available.

pO_2_ NR = 20–40 mmHg	D0	D90	D180	D270	D365
S1	39.4	59.1	55.4	28.9	30.2
S2	26.1	21.8	30.6	16.6	20.7
S3	45.7	23	NA	23.7	23.5
S4	NA	38.3	24.4	29.1	25.7
S5	29.1	23.4	36.3	33.3	21.8
S6	45.8	52.7	82	NA	NA

pCO_2_ NR = 41–51 mmHg					
S1	50.2	36	47.2	50.8	50.4
S2	39.9	47.1	45.7	36.3	39.3
S3	37.4	41.4	NA	46.9	41
S4	NA	49.7	52.6	63.3	56.2
S5	59.4	63.9	59	63.1	63
S6	34.9	45.8	41	NA	NA

pH NR = 7.32–7.42					
51	7.34	7.41	7.34	7.34	7.34
S2	7.4	7.35	7.35	7.46	7.39
S3	7.4	7.4	NA	7.39	7.39
S4	NA	7.32	7.3	7.29	7.32
S5	7.32	7.31	7.34	7.32	7.32
S6	7.36	7.3	7.4	NA	NA

CHCO_3_ NR = 22–27					
S1	26.6	22.1	25.2	26.8	26.5
S2	23.9	25.7	24.6	25.2	23.6
S3	22.5	25.2	NA	27.5	24.3
S4	NA	25.2	25.5	30	28.6
S5	30.1	31.3	31.2	32	31.7
S6	19.1	22	NA	NA	NA

SatO_2_ NR = 40–70%					
S1	72.6	93.4	88.3	52.4	57.1
S2	48.5	33.3	53.1	26	29
S3	99.2	43.7	NA	45.1	45.9
S4	NA	70.7	43.1	57.1	46
S5	30.1	31.3	31.2	32	31.7
S6	81.7	84.9	96	NA	NA
